# Individualized medicine – ethical, economical and historical perspectives

**DOI:** 10.1186/1878-5085-5-S1-A17

**Published:** 2014-02-11

**Authors:** Tobias Fischer, Martin Langanke, Paul Marschall, Susanne Michl

**Affiliations:** 1 Ernst-Moritz-Arndt University, Greifswald, Germany

## 

In the context of the joint project GANI_MED (Greifswald Approach to Individualized Medicine) certain disease phenotypes and biomarker candidates are to be identified with the help of association studies. Thus, GANI_MED aims at contributing to the establishment of routines, improving the implementation of PPPM in the clinical area. A unique characteristic of GANI_MED is the integration of bioethics, history of medicine and health economics throughout the project. The volume starts with a definition of the term “Individualized Medicine” (IM) which is resilient in terms of academic science studies. This will be followed by a chapter that focuses on the socio-cultural backgrounds of IM from the perspective of history of medicine. It will show that IM is also deeply rooted in broader societal developments: the endeavor to extract more and more socio-biological health information from populations in order to detect risk factors and to get a valid data base for conceptualizing efficient curative and preventive interventions on an individual and population-based level. The next chapter will provide deeper insights into medical-analytical research in the framework of GANI_MED: metabolome-related biomarker research, non-pharmaceutical interventions (the example of immunoadsorption) and pharmacogenetics. Chapter 5 will deal with conceptual-ethical questions and critiques of IM and wants to prove to what extent these critiques are justifiable. It also deals with the primary question of IM: do individual risks, that can be detected, play a role for the demand for personal and joint responsibility in a health care system which is shaped by solidarity. A subchapter will deal with the question to what extend IM will face social resistance if its promises of healing can only be formulated against the task of a good, free and self-determined life in general. Beside conceptual questions, also application-orientated problems with regard to research ethics of IM have been investigated within GANI_MED. This includes the Informed Consent architecture which was developed for the question, how participants can be included into such clinical-epidemiological studies as well as considerations about the frequency and processing of incidental findings. The concluding chapter will focus on the questions related to the evaluation of health economics: IM has the potential to alter the rules, institutions and regulations of the health care sector. However, there are major barriers preventing the key-stakeholders to adopt this new approach to medicine. Based on reliable methods of health economic evaluation the relation between the effectiveness of some PPPM-based therapies and the corresponding costs are calculated. This ratio can be regarded as a guiding criterion for decision makers in the health care system for allocating scare resources. The conclusion of the volume will be a chapter in which the most important results from historical, ethical and health-economical single-case study will be summed up.

